# Sister Mary Joseph's Nodule as a Cutaneous Expression of Metastatic Pancreatic Adenocarcinoma

**DOI:** 10.7759/cureus.69944

**Published:** 2024-09-22

**Authors:** Michelle K Custer, Stephen J Lock, Katrin Klemm

**Affiliations:** 1 Dermatology, Edward Via College of Osteopathic Medicine, Auburn, USA; 2 General Surgery, East Alabama Medical Center, Opelika, USA; 3 Pathology, East Alabama Medical Center, Opelika, USA

**Keywords:** abdominal malignancy, advanced pancreatic cancer, cutaneous manifestations of systemic disease, metastatic pancreatic adenocarcinoma, sister mary joseph’s nodule, umbilical discharge, umbilical nodule

## Abstract

A Sister Mary Joseph’s nodule (SMJN) is characterized by a palpable umbilical nodule and is often a clinical indicator of the metastasis of an advanced abdominal or pelvic malignancy. Observing the cutaneous manifestation of an abdomino-pelvic malignancy is a relatively rare phenomenon due to the appearance of visible changes in the later stages of the disease. With the pancreas being a less common primary tumor site for SMJN, this case report describes a 57-year-old male diagnosed with metastatic pancreatic adenocarcinoma with a SMJN. With a history of alcohol use disorder and alcohol pancreatitis, this patient presented to the Emergency Department with worsening abdominal pain and vomiting. A computed tomography (CT) scan with intravenous (IV) contrast revealed a mass in the patient’s rectum and a lobulated mass traversing the anterior abdominal wall, which extended into the umbilicus. A physical exam revealed the presence of an umbilical lesion. A biopsy of the umbilical lesion revealed metastatic adenocarcinoma that favored pancreaticobiliary origin. The results of the umbilical biopsy and CT imaging established the diagnosis of SMJN secondary to pancreatic cancer metastasis to the umbilicus.

## Introduction

Sister Mary Joseph’s nodule (SMJN) is named in remembrance of Sister Mary Joseph (1856-1939), who surgically assisted Dr. William Mayo, a founder of the Mayo Clinic [1]. Being an astute surgical assistant, she consistently observed that an advanced abdominal or pelvic malignancy was present when there was a palpable mass near the patient’s umbilicus [1]. SMJN refers to the presence of a palpable umbilical lump, which can be associated with the metastasis of abdominal and pelvic malignancies [2].

The nodule usually presents as firm, ulcerated, and vascular in appearance [2]. One study reported that 83% of all umbilical tumors are due to metastases [3]. The cutaneous manifestation of an advanced intra-abdominal malignancy is a rare phenomenon, occurring in 1-3% of all abdomino-pelvic malignancies, which is described to be a clinical indication of an aggressive or recurring disease and is associated with poor patient outcomes with an average survival of 10-12 months following confirmation of SMJN [4]. Patients with a SMJN may present with symptoms such as epigastric pain, weight loss, and abdominal enlargement [3].

Noting the aforementioned symptoms associated with an intra-abdominal malignancy, as well as the presence of an umbilical nodule, may assist the clinician in determining a probable cause of the mass. However, it is informative to biopsy the nodule to diagnose the patient. The most common primary neoplasm sites of SMJN include the stomach, ovaries, and colon [5]. Being an uncommon primary neoplasm site for SMJN, the pancreas accounts for nearly 6% of malignant umbilical nodules [6]. The exact process by which the primary tumor spreads to the umbilicus is not well understood, but several explanations include metastasis through the lymphatic channels, vasculature, medial umbilical ligament, or contiguously throughout the body [7].

Because the presence of a SMJN usually presents widespread disease, palliative treatment is typically involved [8]. Other possible treatments include radiotherapy and wide excision surgery [4]. One study demonstrated that patients treated with surgery in combination with adjunctive therapy lived 10.2 months longer than patients treated with surgery alone [9]. In this report, a 57-year-old male diagnosed with metastatic pancreatic adenocarcinoma presenting with a SMJN is described. We also emphasize the importance of routine physical examinations to identify internal malignancies that may present as a SMJN.

## Case presentation

A 57-year-old male patient with a history of alcohol use disorder, alcoholic pancreatitis, and chronic back pain presented to the emergency department with a two-week history of diffuse, worsening abdominal pain, vomiting, constipation, shortness of breath, and unintentional weight loss. During the evaluation of the patient’s abdominal pain, a physical exam revealed the presence of an ulcerated umbilical lesion, which was firm, tender to palpation, lobulated with irregular borders, and produced blood-tinged discharge.

**Figure 1:**
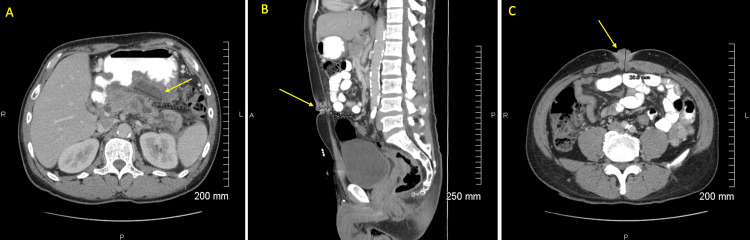
Computed tomography of the abdomen and pelvis with intravenous contrast. (A) New fluid collection between the body of the pancreas and the lesser curvature of the stomach. (B) and (C) Bilobed-shaped solid-appearing lesion at the umbilicus, consistent with Sister Mary Joseph's nodule.

As noted in Figure 1A, a previous computed tomography (CT) scan with intravenous (IV) contrast of the abdomen and pelvis displayed dilation of the pancreatic duct along with a new fluid collection between the body of the pancreas and the lesser curvature of the stomach. A repeat CT scan of the abdomen and pelvis with IV contrast, also shown in Figure 1B and Figure 1C, was performed, which revealed a mass in the patient’s rectum, two mesenteric masses adjacent to the tip of the appendix, and a lobulated mass traversing the anterior abdominal wall, which extended into the umbilicus.

**Figure 2:**
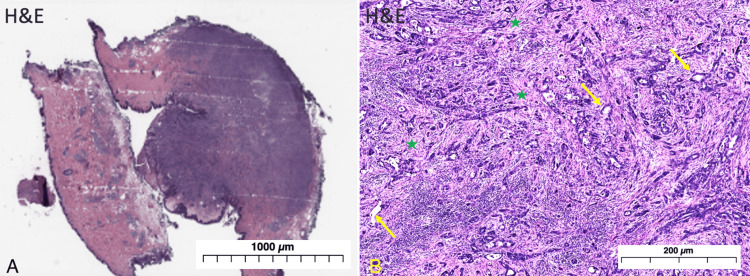
Microscopic examination of the excised umbilical mass. (A) 2x hematoxylin and eosin (H&E) stain of excised umbilical mass. (B) 10x H&E stain of excised umbilical mass displaying glandular formation (yellow arrows) and desmoplastic stroma (green stars) consistent with metastatic deposits of adenocarcinoma.

In addition to pain management with morphine and ketorolac, the patient was administered a vancomycin injection prior to undergoing a biopsy of the umbilical lesion. The results of the biopsy, as pictured in Figure 2, revealed metastatic adenocarcinoma due to glandular formation, desmoplastic stroma, high nucleus-to-cytoplasm ratio, and increased mitotic activity. 

**Figure 3:**
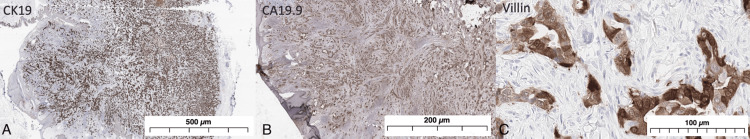
Immunoperoxidase staining of the excised umbilical mass. The neoplastic cells are immunoreactive with antibodies directed against (A) Cytokeratin 19 (CK19) stain shown in a 4X image, (B) cancer antigen 19.9 (CA19.9) shown in a 10x image, and (C) villin shown in a 20x image. This immunoprofile favors a pancreaticobiliary origin.

Additionally, the immunoprofile of the biopsy, shown in Figure 3, indicated a pancreaticobiliary origin of the metastatic adenocarcinoma. Immunoperoxidase staining of the excised umbilical mass described the neoplastic cells as immunoreactive with antibodies directed against Cytokeratin 7 (CK7), focally Caudal-type Homeobox 2 (CDX2), Cytokeratin 19 (CK19), Cancer antigen 19.9 (CA 19.9), and villin. The neoplastic cells did not react with antibodies directed against Cytokeratin (CK20), thyroid transcription factor 1 (TTF1), and NK3 Homeobox 1 (NKX3.1).

The results of the umbilical excisional biopsy and CT imaging established the diagnosis of SMJN secondary to pancreatic cancer metastasis to the umbilicus. Ultimately, he was discharged to oncology services to guide future treatment decisions and scheduled for a follow-up with the outpatient internal medicine clinic following initiation of treatment.

## Discussion

SMJN is a rare clinical finding that manifests as a palpable cutaneous mass in the umbilical region and often predicts an underlying internal malignancy with probable distant spread [8]. As the presence of a SMJN may indicate a malignant disease, 42% of SMJNs arise from a primary neoplasm of the abdomen or pelvis [6]. Although the specific mechanism underlying the spread of a primary malignancy is not clearly delineated, metastasis to the umbilical region is thought to be due to malignant or hematogenous travel [7]. More specifically, it is proposed that gastric cancer can metastasize by way of the lymphatic system to the peritoneum and, finally, to the umbilical area [7]. The cutaneous expression of a SMJN is commonly due to primary malignancies of the stomach, colorectal area, and ovaries [5]. Despite pancreatic cancer being an aggressive malignancy, the pancreas is less commonly associated with a SMJN due to its preference to metastasize through the liver or directly to the peritoneum in a dispersed pattern [6]. 

In the present case, the patient presented with symptoms consistent with an abdominal malignancy, such as abdominal pain, vomiting, and shortness of breath [9]. However, differential diagnoses of an umbilical nodule may include umbilical hernia, fibroma, and keloid due to the similarities in the external appearance of malignant nodules [10]. Endometriosis may also be considered due to the possible formation of palpable pelvic nodules and pelvic or abdominal discomfort [10]. Clinical symptoms and imaging modalities will provide additional guidance on proper investigations to identify the underlying disease.

Radiological imaging may reveal a lesion in the periumbilical region, but a biopsy is needed for a definitive diagnosis [11]. Likewise, although this patient’s repeat CT imaging revealed a mass that extended into the umbilicus, an excisional biopsy was required to diagnose the patient with SMJN secondary to metastatic pancreatic adenocarcinoma. Early detection of SMJN is essential for improving patients’ prognosis. Palliative treatment is routinely implicated in patients with a SMJN due to the diagnosis of the underlying disease at an advanced stage and patients' comorbidities [8]. 

It is imperative for medical providers to understand the significance of finding a SMJN during physical examination and to urgently investigate its etiology. Earlier detection of SMJN will allow for prompt initiation of diagnostic studies, such as biopsies and imaging, which will reveal the causal disease or malignancy.

## Conclusions

The presence of a SMJN is a rare clinical finding that often indicates an underlying internal abdominal or pelvic malignancy. With the pancreas being a less common primary tumor site for SMJN, this case report describes a 57-year-old male diagnosed with metastatic pancreatic adenocarcinoma with a SMJN. Due to the various potential causes of an umbilical nodule, it is critical to consider the patient’s medical history and clinical symptoms to guide further disease investigation. Diagnosing a patient with a SMJN at an earlier stage in their disease course will allow for timely intervention and implementation of treatment plans, which may result in more effective therapy strategies. Ultimately, it is imperative for medical professionals to perform thorough physical examinations and apply clinical knowledge to investigate significant abdominal findings to improve patient outcomes in cases involving SMJN.
